# FixK_2_ Is the Main Transcriptional Activator of *Bradyrhizobium diazoefficiens nosRZDYFLX* Genes in Response to Low Oxygen

**DOI:** 10.3389/fmicb.2017.01621

**Published:** 2017-08-30

**Authors:** María J. Torres, Emilio Bueno, Andrea Jiménez-Leiva, Juan J. Cabrera, Eulogio J. Bedmar, Socorro Mesa, María J. Delgado

**Affiliations:** Department of Soil Microbiology and Symbiotic Systems, Estación Experimental del Zaidín, Consejo Superior de Investigaciones Científicas Granada, Spain

**Keywords:** climate change, denitrification, greenhouse gas, nitrous oxide, nitrous oxide reductase, regulation

## Abstract

The powerful greenhouse gas, nitrous oxide (N_2_O) has a strong potential to drive climate change. Soils are the major source of N_2_O and microbial nitrification and denitrification the main processes involved. The soybean endosymbiont *Bradyrhizobium diazoefficiens* is considered a model to study rhizobial denitrification, which depends on the *napEDABC, nirK, norCBQD*, and *nosRZDYFLX* genes. In this bacterium, the role of the regulatory cascade FixLJ-FixK_2_-NnrR in the expression of *napEDABC, nirK*, and *norCBQD* genes involved in N_2_O synthesis has been previously unraveled. However, much remains to be discovered regarding the regulation of the respiratory N_2_O reductase (N_2_OR), the key enzyme that mitigates N_2_O emissions. In this work, we have demonstrated that *nosRZDYFLX* genes constitute an operon which is transcribed from a major promoter located upstream of the *nosR* gene. Low oxygen was shown to be the main inducer of expression of *nosRZDYFLX* genes and N_2_OR activity, FixK_2_ being the regulatory protein involved in such control. Further, by using an *in vitro* transcription assay with purified FixK_2_ protein and *B. diazoefficiens* RNA polymerase we were able to show that the *nosRZDYFLX* genes are direct targets of FixK_2_.

## Introduction

Nitrous oxide (N_2_O) is a powerful greenhouse gas (GHG) and a major cause of ozone layer depletion with an atmospheric lifetime of 114 years and, based on its radiative capacity, an estimated 300-fold greater potential for global warming compared with that of carbon dioxide (CO_2_). Hence, N_2_O accounts for approximately 10% of total emissions with respect to the impact of each individual GHGs on global warming (Intergovernmental Panel on Climate Change [IPCC], 2014). Due to its environmental impact, a better understanding of the pathways implicated in the generation and consumption of N_2_O has received great interest (Thomson et al., 2012).

Despite the existence of multiple pathways for N_2_O generation in soils such as nitrifier denitrification, nitrite oxidation, heterotrophic denitrification, ammonia oxidation, anaerobic ammonium oxidation (anammox) and dissimilatory nitrate reduction to ammonium (DNRA), it is generally assumed that nitrification and denitrification are the principal processes that contribute to the emissions of N_2_O from terrestrial ecosystems (for a review see Stein, 2011; Schreiber et al., 2012; Butterbach-Bahl et al., 2014). Denitrification is widespread within the domain of *Bacteria* being dominant within *Proteobacteria* (Shapleigh, 2006). However, it has been evinced that some archaea (Treusch et al., 2005) and fungi (Takaya, 2002; Prendergast-Miller et al., 2011) may also denitrify. Most of the studies about denitrification have been focused on Gram-negative bacteria that occupy terrestrial niches, using the alpha-proteobacterium *Paracoccus* (*Pa*.) *denitrificans* as well as the gamma-proteobacteria *Pseudomonas* (*Ps.*) *stutzeri* and *Ps. aeruginosa* as model organisms (Zumft, 1997). The reactions of denitrification are catalyzed by periplasmic (Nap) or membrane-bound (Nar) nitrate reductase, nitrite reductases (NirK/NirS), nitric oxide (NO) reductases (cNor, qNor, or Cu_A_Nor) and nitrous oxide reductase (N_2_OR) encoded by *nap*/*nar, nirK*/*nirS, nor*, and *nos* genes, respectively. The physiological, biochemical and molecular aspects of denitrification have been covered by a collection of reviews published elsewhere (Zumft, 1997; van Spanning et al., 2005, 2007; Kraft et al., 2011; Richardson, 2011; Bueno et al., 2012).

In contrast to the numerous sources of N_2_O, nitrous oxide reductase (NosZ) is the only known biological enzyme involved in its removal by reduction to N_2_ (reviewed by Thomson et al., 2012). A new cluster of atypical *nosZ* genes, designated clade II, have been recently identified (Sanford et al., 2012; Jones et al., 2013) which are also present in genomes lacking the *nirS* and/or *nirK* gene. This suggests that non-denitrifiers also contribute to N_2_O removal (Jones et al., 2013).

Nitrous oxide reductase is a homodimer with molecular weight of 120-160 kDa, a copper content of ∼12 Cu atoms, and a sulfide content of ∼2 S^2-^ ions per dimer (Rasmussen et al., 2000). The enzyme contains two copper sites: Cu_A_, and Cu_Z_, a tetranuclear μ4-sulfide-bridged cluster liganded by seven histidine residues, which has been proposed to be the active center for N_2_O reduction. The expression, maturation, and maintenance of the NosZ catalytic subunit require several other auxiliary proteins (Zumft, 2005) being all encoded together by a typical gene cluster that contains six genes (*nosRZDFYL*). This core cluster is, in some cases, associated with an additional gene, *nosX* (reviewed by Zumft and Kroneck, 2007). Mutation analyses demonstrated that NosDFY or NosL are involved in the maturation of the NosZ Cu_Z_, but not in the biogenesis of the Cu_A_ site (reviewed by Zumft and Kroneck, 2007; van Spanning, 2011). NosR and NosX do not participate in Cu_Z_ biogenesis but do play a role in N_2_O reduction *in vivo* altering the state of the Cu_Z_ site during turnover and supporting the catalytic activity of NosZ (Wunsch and Zumft, 2005). NosR, apart from its putative role as electron donor to NosZ, might also act as a regulator, since it is needed for *Ps. stutzeri nosZ* and *nosD* transcription (Honisch and Zumft, 2003).

Low O_2_ conditions and NO have been suggested as the main signal molecules for induction of *nos* genes expression (reviewed by Zumft and Kroneck, 2007). Both signals are perceived and transduced via transcriptional regulators belonging to the cyclic AMP receptor protein (CRP)/fumarate and nitrate reductase (FNR) superfamily. This family carries diverse mnemonics, such as ANR, DNR, NNR, NnrR, FNR or FixK but all refer to the same type of regulatory protein with similar domain structure. Proteins that form part of the DNR clade such as DNR/DnrD/NNR from *Ps. aeruginosa, Ps. stutzeri*, and *Pa. denitrificans*, respectively (van Spanning et al., 1999; Vollack and Zumft, 2001; Zumft and Kroneck, 2007; Arai et al., 2013), control *nos* genes expression in response to NO, while low oxygen is perceived by [4Fe-4S]^2+^ cluster-containing FNR- and FnrP-type proteins such as *Pa. denitrificans* FnrP (Bergaust et al., 2012) or *Ps*. *aeruginosa* ANR (Trunk et al., 2010).

*Bradyrhizobium diazoefficiens* (Delamuta et al., 2013; formerly *B. japonicum*), the endosymbiont of soybeans, possesses the ability to denitrify under both free-living and symbiotic lifestyles. In *B. diazoefficiens* the denitrification process depends on the *napEDABC, nirK, norCBQD*, and *nosRZDYFLX* genes, coding for Nap, copper-containing NirK, *c*-type Nor and the N_2_OR, respectively (Velasco et al., 2001, 2004; Mesa et al., 2002; Delgado et al., 2003; Bedmar et al., 2005).

Expression of *B. diazoefficiens* denitrification genes required low oxygen tension and in the case of *norCBQD* genes the presence of NO is also needed (Bueno et al., 2017). In this bacterium, perception and transduction of the ‘low-oxygen’ signal are mediated by a complex network comprising two interconnected regulatory cascades, the FixLJ–FixK_2_–NnrR and the RegSR–NifA (Sciotti et al., 2003). In the latter cascade, an oxygen concentration at or below 0.5% is required for activation of the oxygen-sensitive NifA protein and subsequent induction of essential nitrogen fixation genes (Sciotti et al., 2003). Under anoxic conditions in the presence of NO_3_^-^, NifA is also necessary for the maximal expression of *napE-lacZ, nirK-lacZ*, and *norC-lacZ* fusions (Bueno et al., 2010). Moreover, global transcription analyses of a *regR* mutant in comparison to the wild-type (WT), both grown in anoxic denitrifying conditions showed that RegR is also involved in the regulation of *B. diazoefficiens norCBQD* and *nosRZDYFLX* genes (Torres et al., 2014).

In contrast as reported for the RegSR-NifA cascade, activation of expression of the FixLJ-FixK_2_-NnrR-dependent targets requires a moderate decrease in the oxygen concentration in the gas phase (≤5%), where the haem-based sensory kinase FixL senses the ‘low-oxygen’ signal, phosphorylates itself and transfers the phosphoryl group to the FixJ response regulator. Then, FixJ activates transcription of the *fixK_2_* gene, encoding the FixK_2_ protein, a CRP/FNR-like transcriptional regulator. FixK_2_ induces, in turn, expression of the *napEDABC, nirK*, and *norCBQD* denitrification genes involved in N_2_O production (Velasco et al., 2001; Mesa et al., 2002; Robles et al., 2006) as well as other regulatory genes [e.g., *rpoN*_1_, *fixK*_1_, and *nnrR*; (Nellen-Anthamatten et al., 1998; Mesa et al., 2003, 2008)]. The latter, the CRP/FNR-type NnrR protein adds an additional control level to the FixLJ-FixK_2_ cascade integrating the NOx signal necessary for induction of *norCBQD* genes expression (Mesa et al., 2003; Bueno et al., 2017). Within the CRP/FNR family, FixK_2_ belongs to the FixK subgroup, whose members, in contrast to the O_2_-sensitive proteins *Ps. aeruginosa* ANR and *Pa. denitrificans* FnrP, lack the cysteine motif required to bind an [4Fe-4S]^2+^ cluster (reviewed in Korner et al., 2003; Mesa et al., 2006). Particularly, FixK_2_ activity is subjected to posttranslational control by oxidation of its singular cysteine residue at position 183 (Mesa et al., 2009). *B. diazoefficiens* NnrR forms part of the NnrR clade, proteins that cover a similar function to the one defined for DNR-type proteins on the control of denitrification genes expression in response to NO (Bueno et al., 2017). Recently, we observed that *B. diazoefficiens napEDABC, nirK*, and *norCBQD* promoters exhibited differences with regard to their dependence on low oxygen (microoxia), NOx, and the regulatory proteins FixK_2_ and NnrR. While microoxic conditions were sufficient to induce expression of *napEDABC* and *nirK* genes and this control directly depends on FixK_2_, *norCBQD* genes expression depends on NO, NnrR being the candidate that directly interacts with *norCBQD* promoter (Bueno et al., 2017).

As described for other CRP/FNR members, FixK_2_ acts as a dimeric form which binds to a twofold symmetric DNA sequence present at distinct distances within the promoter region of regulated genes (Browning and Busby, 2004). Specifically, the FixK_2_ box corresponds to TTG(A/C)-N_6_-(T/G)CAA (Bonnet et al., 2013), which matches reasonably well with the previously described consensus binding site for FixK-type proteins (TTGA-N_6_-TCAA) (Fischer, 1994; Dufour et al., 2010).

While substantial progress has been made on the external signals (microxia and NO) and the manner by which the FixK_2_ and NnrR proteins control the expression of *B. diazoefficiens napEDABC, nirK*, and *norCBQD* genes involved in N_2_O synthesis, the regulation of *nosRZDYFLX* genes involved in N_2_O reduction to N_2_, the key step to N_2_O mitigation, has been very poorly explored in this bacterium. In the present work, we show the transcriptional arrangement of the *nosRZDYFLX* genes in *B. diazoefficiens*. We also expanded the knowledge on *nosRZDYFLX* regulation by studying the involvement of low oxygen, and NOx in *nos* expression as well as the role of FixK_2_ and NnrR regulatory proteins in this control. By using *in vitro* transcription (IVT) activation assays we demonstrated, for first time, that the *nosRZDYFLX* genes are direct targets of FixK_2_.

## Materials and Methods

### Bacterial Strains, Media, and Growth Conditions

Bacterial strains used in this work are compiled in **Table 1**. *Escherichia coli* cells were cultivated in Luria Bertani medium (Miller, 1972) at 37°C. When needed, antibiotics were used at the following concentrations (in μg/ml): ampicillin, 200; kanamycin, 30; spectinomycin, 25; streptomycin, 25; tetracycline, 10.

**Table 1:** Bacterial strains and plasmids used in this study.

Strains and plasmids	Relevant description	Source of reference
**Strains**		
*E. coli*		
DH5α	*supE44*Δ*lacU*169 (ϕ80 *lacZ*ΔM15) *hsdR17 recA1 gyrA96 thi-1 relA1*	Bethesda Research Laboratories Inc., Gaithersburg, MD, United States.
S17.1	Tp^r^ Sm^r^ Spc^r^ *thi, pro*, recA, *hsdR, hsdM*, RP4Tc::Mu, Km::Tn7	Simon et al., 1983
BL21 (DE3)	F^-^ *opmT hsdS*_B_(rB^-^ mB^-^) *gal dcm* (DE3)	Novagen Inc.
*B. diazoefficiens*		
USDA110	Cm^r^ wild-type	United States Department of Agriculture, Beltsville, MD, United States
110*spc*4	Cm^r^ Sp^r^ wild-type, a spectinomycin-resistant derivative of USDA110	Regensburger and Hennecke, 1983
GRPA1	Cm^r^ Spc^r^ Sm^r^ *napA*::Ω	Delgado et al., 2003
GRK308	Cm^r^ Spc^r^ Sm^r^ *nirK*::Ω	Velasco et al., 2001
9043	Cm^r^ Spc^r^ Sm^r^ Δ*fixK_2_*::Ω	Nellen-Anthamatten et al., 1998
8678	Cm^r^ Spc^r^ Km^r^ Δ*nnrR*::*aphII*	Mesa et al., 2003
GRZ3035	Cm^r^ Spc^r^ Sm^r^ *nosZ*::Ω	Velasco et al., 2004
110*spc*4-BG0301	Cm^r^ Sp^r^ Tc^r^ *nosR*-*lacZ* chromosomally integrated into 110*spc*4	This work
110*spc*4-BG0302	Cm^r^ Sp^r^ Tc^r^ *nosZ*-*lacZ* chromosomally integrated into 110*spc*4	This work
110*spc*4-BG0303	Cm^r^ Sp^r^ Tc^r^ *nosD*-*lacZ* chromosomally integrated into 110*spc*4	This work
110*spc*4-BG0304	Cm^r^ Sp^r^ Tc^r^ *nosR*-*lacZ* chromosomally integrated into 110*spc*4 full FixK_2_-like box	This work
110*spc*4-BG0305	Cm^r^ Sp^r^ Tc^r^ *nosR*-*lacZ* chromosomally integrated into 110*spc*4 half FixK_2_-like box	This work
110*spc*4-BG0306	Cm^r^ Sp^r^ Tc^r^ *nosR*-*lacZ* chromosomally integrated into 110*spc*4 no FixK_2_-like box	This work
GRPA1-BG0301	Cm^r^ Sp^r^ Sm^r^ Tc^r^ *nosR*-*lacZ* chromosomally integrated into GRPA1	This work
GRK308-BG0301	Cm^r^ Sp^r^ Sm^r^ Tc^r^ *nosR*-*lacZ* chromosomally integrated into GRK308	This work
9043-BG0301	Cm^r^ Sp^r^ Sm^r^ Tc^r^ *nosR*-*lacZ* chromosomally integrated into 9043	This work
8678-BG0301	Cm^r^ Sp^r^ Tc^r^ *nosR-lacZ* chromosomally integrated into 8678	This work
**Plasmids**		
pSUP3535	Tc^r^ transcriptional *lacZ* fusion suicide vector	Mesa et al., 2003
pRJ9519	Ap^r^ [pBluescript SK(+) 308-bp BstXI-KpnI fragment containing the *B. diazoefficiens rrn* terminator cloned into the HincII and KpnI sites]	Beck et al., 1997
pBG0301	Tc^r^ (pSUP3535) *nosR* 5′ region on a 558-bp EcoRI-PstI fragment	This work
pBG0302	Tc^r^ (pSUP3535) *nosZ* 5′ region on a 1024-bp EcoRI fragment	This work
pBG0303	Tc^r^ (pSUP3535) *nosZ* 5′ region on a 875-bp EcoRI fragment	This work
pBG0304	Tc^r^ (pSUP3535) *nosR* 5′ region containing the intact FixK_2_-like box on a 224-bp EcoRI fragment	This work
pBG0305	Tc^r^ (pSUP3535) *nosR* 5′ region containing a partial FixK_2_-like box on a 218-bp EcoRI fragment	This work
pBG0306	Tc^r^ (pSUP3535) *nosR* 5′ region lacking the FixK_2_-like box on a 168-bp EcoRI fragment	This work
pDB4020	Ap^r^ (pRJ9519) *nosRZDFYLX* promoter on a 486-bp XbaI-EcoRI fragment	This work
pRJ0004	Km^r^ [pET-24c(+)] with a 701-bp NdeI/NotI fragment encodingC183S-FixK_2_-His_6_	Bonnet et al., 2013

*Bradyrhizobium diazoefficiens* cells were cultured oxically and microoxically basically as described earlier (Bueno et al., 2017). While Peptone-Salts-Yeast extract (PSY) medium (Regensburger and Hennecke, 1983; Mesa et al., 2008) was employed in routine oxic cultures, Yeast Extract-Mannitol (YEM) medium (Daniel and Appleby, 1972) was used as standard medium in our experiments. After growth under oxic conditions in PSY medium, cells were collected by centrifugation (8.000 *g* for 10 min at 4°C), and washed twice with YEM medium. Next, washed cells were used to inoculate, at a 600 nm optical density (OD_600_) of 0.2, 17 ml or 500 ml rubber stoppered tubes or Erlenmeyer flasks containing 3 ml or 150 ml of YEM medium amended or not with 10 mM KNO_3_, respectively. Next, cells were incubated for 24 h under low oxygen conditions, either at initial 0.5% O_2_ or at 2% O_2_ (in this case the headspace was exchanged every 8–16 h). The latter conditions were chosen to study the specific control of the FixK_2_ and NnrR regulatory proteins. To analyze the effect of the different NOx, microoxically incubated cells were subsequently exposed for 5 h to 10 mM KNO_3_, 500 μM NaNO_2_, 50 μM NO (from a saturated NO solution [1.91 mM at 20°C]), and 0.15% (30 mM) N_2_O. 10 μM or 100 μM of the NO-scavenger cPTIO [2-(4-Carboxyphenyl)-4,4,5,5-tetramethylimidazoline-1-oxyl-3-oxide; carboxy-PTIO potassium salt; Sigma] was added from the beginning to the WT and Δ*nnrR* strain cultures grown microoxically (2% O_2_) in the presence of 10 mM KNO_3_ for 24 h, in order to analyze the effect of removing the excess of NO on the expression of *nosR-lacZ* or N_2_OR activity, respectively. Antibiotics were added to the *B. diazoefficiens* cultures at the following concentrations (μg/ml); chloramphenicol, 20; streptomycin, 200; kanamycin, 200; tetracycline, 100 (solid cultures), 25 (liquid cultures); spectinomycin, 200.

### Plasmids and Bacterial Strains Construction

Plasmids used in this study are listed in **Table 1**. Primer sequences in this work are compiled in Supplementary Table S1. For construction of transcriptional reporter fusion plasmids, 5′ DNA fragments for the *nosR* (558; 132; 128 and 75 bp), *nosZ* (1024 bp) and *nosD* (875 pb) promoter regions were amplified using primers’ pair a1/PnosR.r, PnosRfull.f/PnosR.r, PnosRhalf.f/PnosR.r, PnosRno.f/PnosR.r, PnosZ.f/PnosZ.r and c1/c2, respectively (Supplementary Table S1). The PCR products were then individually ligated into the pGEM^®^-T vector (Promega), digested with EcoRI or EcoRI-PstI and cloned into the *lacZ* fusion suicide vector pSUP3535 (Mesa et al., 2003), to yield plasmids pBG0301, pBG0304, pBG0305, pBG0306, pBG0302, and pBG0303, respectively (see **Table 1** for details). The correct orientation of the inserts was verified by sequencing. Plasmids pBG0301, pBG0302, pBG0303, pBG0304, pBG0305, and pBG0306 were integrated by homologous recombination into the chromosome of WT *B. diazoefficiens* 110*spc*4, yielding strains 110*spc*4-BG0301, 110*spc*4-BG0302, 110*spc*4-BG0303, 110*spc*4-BG0304, 110*spc*4-BG0305, 110*spc*4-BG0306. Plasmid pBG0301 was also integrated into the chromosome of *napA* (GRAP1), *nirK* (GRK308), *fixK_2_* (9043), and *nnrR* (8678) mutants, yielding strains GRPA1-BG0301, GRK308-BG0301, 9043-BG0301, and 8678-BG0301, respectively (**Table 1**). Correct recombination into the chromosome of the corresponding recipient strain was checked by PCR analyses.

The plasmid used as transcription template was based on the plasmid pRJ9519 which contains a *B. diazoefficiens rrn* transcriptional terminator (Beck et al., 1997). The *nosRZDFYLX* promoter was PCR-amplified with nosR_For_Transc and nosR_Rev_Transc primers, subsequently restricted with XbaI and EcoRI, and finally cloned as a 486-bp fragment into pRJ9519, yielding plasmid pDB4020. The correct nucleotide sequence was confirmed by sequencing.

### Analysis of *nosRZDFYLX* Genes Co-transcription by RT-PCR

End-point reverse transcription-polymerase chain reaction (RT-PCR) was performed to investigate the transcriptional architecture of *nosRZDFYLX* genes. First, *B. diazoefficiens* cells were grown under 0.5% initial O_2_ concentration to an OD_600_ of ∼0.4 in YEM medium supplemented with 10 mM KNO_3_. Cell harvest and isolation of total RNA were done as described previously (Hauser et al., 2007; Lindemann et al., 2007; Mesa et al., 2008). First strand cDNA synthesis was performed with the SuperScript II reverse transcriptase (Invitrogen) according to the supplier’s guidelines, using 1 μg of total RNA and primers c2 and g2 that hybridize in the complementary sequence of *nosD* and *nosX* genes. The obtained cDNA was next used for amplification of putative intergenic regions between *nosR* and *nosX* (blr0314-blr0320) using primers’ pairs labeled as b1/b2-to-g1/g2 and flanking regions using primers’ pair labeled as a1/a2 and h1/h2 (Supplementary Table S1), essentially as described by Sambrook and Russell (2001). In negative controls, reverse transcriptase was omitted in the reaction. Positive control PCR reactions were performed with *B. diazoefficiens* genomic DNA as template.

### 5′ RACE of *B. diazoefficiens nosRZDFLYX* Genes

The transcription start sites of *nos* genes were determined with the RACE (Rapid Amplification of cDNA Ends) method as described by Sambrook and Russell (2001). Cell cultivation and harvest as well as total RNA isolation were carried out as described above for the RT-PCR experiments. First strand cDNA synthesis was performed with the SuperScript II reverse transcriptase (Invitrogen) according to the supplier’s guidelines, using 0.8 μg of total RNA and primer SP1_nosR. After the reaction, dNTPs and primers were removed with the GeneJET PCR Purification Kit (Thermo Fisher Scientific) and products were eluted in 15 μl of 10 mM Tris-HCl, pH 8.5. Poli-A tails were added to 5′ end of cDNAs with the terminal deoxynucleotidyl transferase (Thermo Fisher Scientific) and final products were diluted with purified water to final volume of 1 ml. Amplification reactions were carried out with primers (dT)_17_-adaptor-primer, adaptor-primer and SP2_ nosR primers using the following PCR program: 95°C for 5 min; (95°C for 30 s; 48°C for 30 s; 72°C for 45 s) × 5 cycles; (95°C for 30 s; 55°C for 30 s; 72°C for 45 s) × 30 cycles; 72°C for 10 min and hold at 4°C. DNA libraries were constructed by cloning the PCR products into pGEM-T easy vector (Promega). Plasmid DNA of individual clones was purified with QIAprep Spin Miniprep Kit (Qiagen) and Sanger sequenced using SP6 as primer. Transcription start sites were identified as the first nucleotide sequenced after the poly-A sequence.

### Analysis of *nosRZDFYLX* Gene Expression by qRT-PCR

Expression of *nosR* was also analyzed by qRT-PCR using an iQTM5 Optical System (Bio-Rad, Foster City, CA, United States). *B. diazoefficiens* WT and *napA, nirK, fixK_2_*, and *nnrR* mutant strains were grown in YEM medium amended with 10 mM NO_3_^-^ under initial 0.5% O_2_ (WT, *napA* and *nirK* mutant strains) or 2% O_2_ (WT, *fixK_2_* and *nnrR* mutant strains) for 24 h. Cell harvest, isolation of total RNA and cDNA synthesis were done as described previously (Hauser et al., 2007; Lindemann et al., 2007; Mesa et al., 2008). Primers for the PCR reactions (nosR_qRT_PCR_F/ nosR_qRT_PCR_R; Supplementary Table S1) were designed with the Clone Manager Suite 9 software to have melting temperatures between 57 and 62°C and generate PCR products of 50–100 bp. Each PCR reaction contained 9.5 μl of iQTM SYBR Green Supermix (Bio-Rad), 2 μM (final concentration) of individual primers and appropriate dilutions of different cDNA samples in a total volume of 19 μl. Reactions were run in triplicate. Melting curves were generated to verify the specificity of the amplification. Relative changes in gene expression were calculated as described by Pfaffl (2001). Expression of the 16S *rrn* gene was used as reference for normalization (primers 16S_qRT_For and 16S_qRT_Rev; Supplementary Table S1).

### β-Galactosidase Activity Determination

β-galactosidase activity was determined by using permeabilised cells from at least three independently grown cultures assayed in triplicate essentially as previously described (Cabrera et al., 2016). Specific activities were calculated in Miller units (Miller, 1972).

### N_2_OR Activity

*B. diazoefficiens* cells were incubated microoxically (2% O_2_) for 24 h in YEM medium supplemented or not with 10 mM NO_3_^-^. In the latter conditions, parallel replicates were also exposed to 100 μM of the NO-scavenger cPTIO. Next, cells were washed three times with YEM medium and 30 μl gaseous aliquots of 2% N_2_O in 98% N_2_ (0.15% N_2_O final concentration in the headspace) were injected into the rubber stoppered Erlenmeyer flasks. After 5 h of incubation at 30°C at 185 rpm, gas-liquid phase equilibration was reached and 500-μl gaseous aliquots were taken from the headspace to analyze N_2_O consumption by gas chromatography as described previously (Tortosa et al., 2015).

The protein concentration was estimated using the Bradford method (Bio-Rad Laboratories) with a standard curve constructed with varying bovine serum albumin (BSA) concentrations. N_2_OR activity was determined by using cells from at least three independently biological grown cultures.

### Immunoblot Analyses

*B. diazoefficiens* cells incubated micooxically (2% O_2_) in YEM medium in the presence or absence of 10 mM NO_3_^-^ for 24 h, were harvested and the soluble fraction of the cells was obtained by following the protocol previously described by Delgado et al. (2003). The resulting membrane pellet was discarded and the supernatant, containing the soluble fraction, was concentrated to about 100 μl by using AmiconR Ultra-2 centrifugal filter devices (Millipore) and stored at -20°C until their use. Protein concentration was estimated as described above.

For immunodetection of NosZ, protein samples (10 μg of the soluble fraction) were separated by 12% SDS-polyacrylamide gel electrophoresis (PAGE) as described by Laemmli (1970). Then, proteins were transferred to nylon or PVDF membranes (Millipore). The membrane was then incubated in blocking buffer [5% non-fat dry milk in TTBS buffer containing 50 mM Tris-HCl pH 7.5, 0.15 mM NaCl and 0.1% Tween 20], with overnight shaking at 4°C. Afterward, the membrane was then washed with TTBS buffer (four times for 10 min each), before being incubated in 10 ml of blocking buffer containing 1/1000 (v/v) antibody dilution (anti-NosZ of *Pa. denitrificans*; Felgate et al., 2012). The membrane was subsequently incubated by shaking gently for 1 h at room temperature (RT). Further, the membrane was then washed with TTBS and incubated for 1 h at RT with a 1/3500 (v/v) dilution of the secondary antibody (sheep anti-IgG: peroxidase antibody produced in donkeys; A3415 Sigma–Aldrich) in blocking buffer. Next the membrane was washed four times with TTBS before adding 500 μl of ECL Select western-blotting detection reagent (GE Healthcare, Amersham) followed by Chemiluminescent signal detection in a Chemidoc XRS (Universal Hood II, Bio-Rad). The Quantity One software (Bio-Rad) was used for image analyses.

### Purification of *B. diazoefficiens* RNA Polymerase

Purification of the *B. diazoefficiens* holoenzyme was carried by using a modified protocol similar to the one described by Beck et al. (1997). 25 g (wet weight) of *B. diazoefficiens* 110*spc*4 cells grown oxically in PSY supplemented with 0.1% arabinose until late exponential phase were used for each purification batch. All purification steps were performed at 4°C. Cells were resuspended in 70 ml of TGED buffer (10 mM Tris-HCl [pH 8.0], 10% glycerol, 1 mM EDTA, 0.1 mM dithiothreitol [DTT]) containing 0.02 M NaCl and 1 mM ABSF and disrupted in a French pressure cell (three passes at 1000 psi). The crude extract was treated with polyethyleneimine to a final concentration of 0.3%. The pellet obtained after centrifugation (15 min; 27,000 × *g*) was washed with TGED buffer (0.2 M NaCl), and protein containing RNAP was washed three times in TGED buffer (0.8 M NaCl). In all recovery steps, the supernatant was collected and precipitated again by adding solid (NH_4_)_2_SO_4_ to 65% final saturation (43 g per 100 ml). The precipitate was collect by centrifugation (30 min; 27,000 × *g*), dissolved in 30 ml of TGED buffer (0.02 M NaCl) and, dialyzed against 1 liter of TGED buffer (0.02 M NaCl). The dialyzed sample was loaded onto an HiTrap Q FF column (GE Healthcare), from which it was eluted by a linear 0.02–1.2 M NaCl gradient. Fractions containing RNAP (as judged by standard transcription assays) were pooled and loaded onto a heparin agarose column (HiTrap Heparin HP; GE Healthcare). Equilibration and elution buffers were similar to those used in the HiTrap Q FF chromatography. Peak fractions contained the RNAP (indicated by general IVT assays performed according Beck et al., 1997) were pooled, concentrated by ultrafiltration (YM30 membrane, Amicon), and dialyzed and stored in TGED buffer (0.02 M NaCl) containing 50% glycerol at -20° or -80°. The purity of the active fractions was tested by SDS-PAGE. Protein concentrations were determined with Bio-Rad assay solution, with BSA as the standard.

### IVT Activation Assay

Multiple-round *in vitro* transcription (IVT) assays were carried out as described previously (Beck et al., 1997; Mesa et al., 2008). Plasmid pDB4020 was used as template to study the capacity of the FixK_2_ protein to initiate transcription from the *nosRZDFYLX* promoter. Expression and purification of an oxidation-insensitive C-terminal Histidine-tagged C183S FixK_2_ protein variant (C183S-FixK_2_-His_6_; Bonnet et al., 2013) were carried out as described in (Mesa et al., 2005). Purified FixK_2_ protein was used at concentrations of 1.25 or 2.5 μM dimer.

Runoff transcripts of 286 and 180 nucleotides produced *in vitro* following the procedure used by Mesa et al. (2005) were used as RNA size markers. Transcripts were visualized with a PhosphorImager and signal intensities were determined with the Bio-Rad Quantity One software (Bio-Rad).

## Results

### Transcriptional Organization of the *B. diazoefficiens nosRZDFYLX* Genes

Analysis of the *nosRZDFYLX* sequence did not reveal any predicted transcriptional termination signals^1^ which is an indication that they might be transcribed as an operon. Overlapping coding regions between *nosR* and *nosZ*, as well as between *nosD, F, Y*, and *L* stop and start codons, suggest translational couplings between *nosRZ* and *nosDFYL*. However, unlike these translational couplings, there is a short intergenic region of 14 nucleotides between *nosZ* and *nosD* and 11 nucleotides between *nosL* and *nosX*.

**FIGURE 1:**
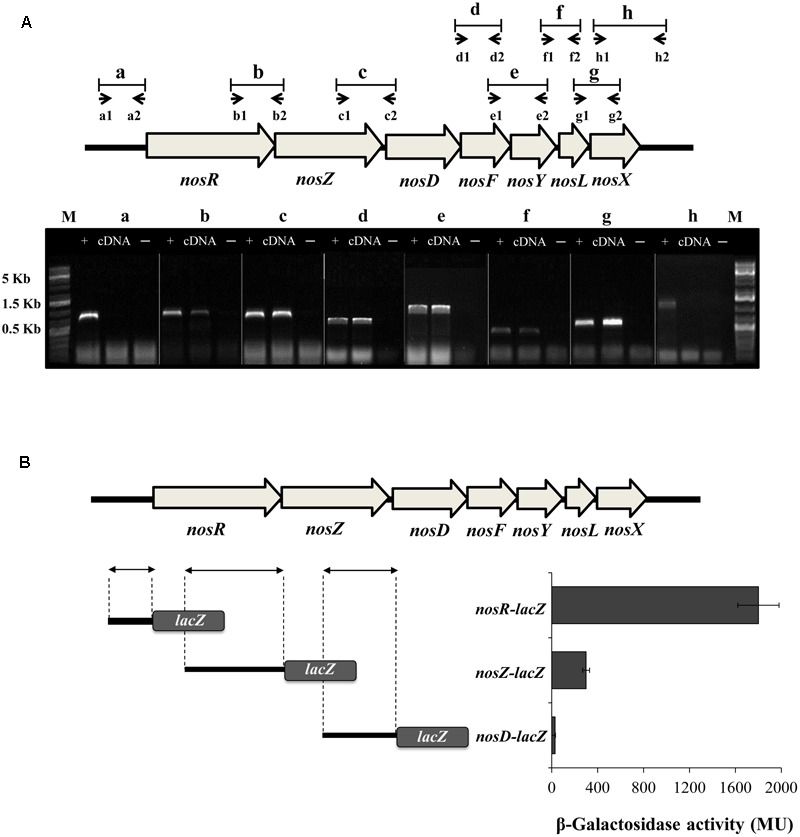
Transcriptional organization of the *B. diazoefficiens nosRZDFYLX* genes. **(A)** Putative intergenic regions probed by RT-PCR are labeled as “b-to-g” and the small arrows below depict positions and orientation of the PCR primers used for each intergenic region. Combinations of primers a1/a2 and h1/h2 were used to amplify “a” and “h” flanking regions. Primer sequences are shown in Supplementary Table S1. RT-PCR products of each primers’ pair combination were loaded on an agarose gel. Total RNA served as the template for cDNA synthesis by using gene specific primers that hybridize in the complementary sequence of *nosD* and *nosX* genes. PCR amplifications using genomic DNA as template (+) or without reverse transcriptase (–) served as positive and negative controls, respectively. The sizes of the marker (M) bands are labeled on the left side. **(B)** β-galactosidase activity from the DNA regions upstream of *nosR, nosZ*, and *nosD* genes fused to the *lacZ* reporter gene (on the right). On the left, the DNA regions fused to *lacZ* are depicted by arrows. In **(A,B)**, *B. diazoefficiens* wild-type (WT) cells were grown for 24 h under low oxygen conditions (initial 0.5% O_2_) with 10 mM KNO_3_. Data expressed as Miller units (MU) represent mean values and error bars from triplicate samples from at least two independent cultures.

In order to investigate the transcriptional architecture of *nosRZDFYLX* genes, end-point RT-PCR was performed to detect intergenic regions between each pair of correlative genes. To ensure that the amplified RT-PCR product was from the template mRNA, each RT-PCR reaction had a negative control (without reverse transcriptase) and a positive control (genomic DNA). First, total RNA was isolated from *B. diazoefficiens* WT cells cultured with initial 0.5% O_2_ concentration in the presence of NO_3_^-^ and subsequently reverse transcribed to cDNA. As shown in **Figure 1A**, specific cDNA products were obtained for intergenic regions designed as b-to-g, but not from those labeled as “a” and “h” corresponding to flanking regions of the *nosRZDFYLX* genes. These findings reveal that *B. diazoefficiens nosRZDFYLX* genes constitute a transcriptional unit, although we cannot discard the presence of additional internal promoters.

To test any potential transcription from the DNA regions upstream of the *nosR, nosZ*, and *nosD* genes, we determined β-Galactosidase activity of chromosomally integrated transcriptional fusions between the DNA regions preceding the annotated *nosR, nosZ, nosD* genes and the reporter gene *lacZ* (**Figure 1B**). After growing *B. diazoefficiens* cells under an initial O_2_ concentration of 0.5% O_2_ in the presence of NO_3_^-^, the highest transcriptional expression was driven from the *nosR*-*lacZ* fusion compared to the *nosZ*-*lacZ* and *nosD-lacZ* fusions (**Figure 1B**). These results strongly suggest that transcription of *nosRZDFYLX* mainly depends on a promoter present in the DNA region upstream of *nosR.* However, although β-galactosidase activity from the *nosZ-lacZ* fusion was sixfold lower to that observed from the *nosR-lacZ* fusion, we cannot exclude the possibility that another internal promoter upstream of *nosZ* might exist.

**FIGURE 2:**
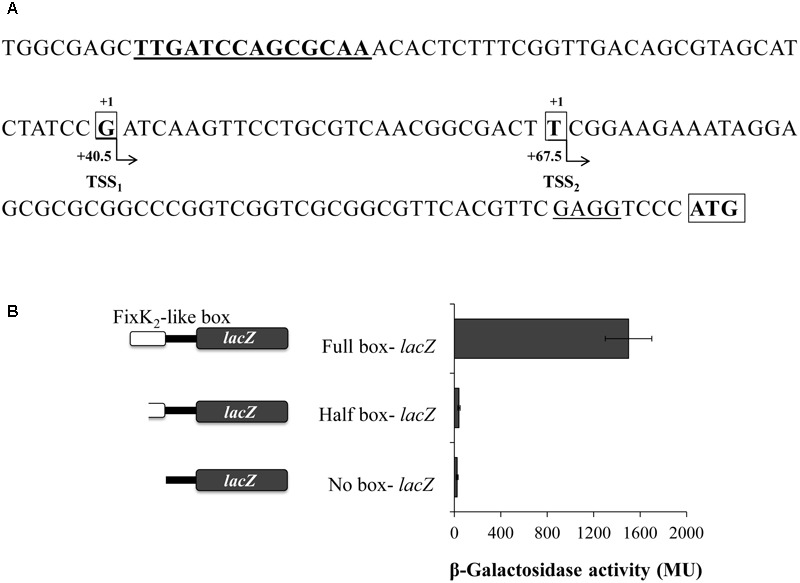
Structure of the *B. diazoefficiens nosR* promoter. **(A)** Sequence and schematic representation of the *nosR* promoter region. Nucleotides corresponding to the transcription start sites (TSS) located at +40.5 and +67.5 bp from the axis of symmetry of the putative FixK_2_-like box (bold and underlined) identified by RACE are shown in bold, marked with “+1” above and highlighted with an open black box. The annotated *nosR* translation start codon (ATG; http://genome.annotation.jp/RhizoBase) is shown in bold with an open black box. A putative ribosome binding site of *nosR* is underlined. **(B)** β-Galactosidase activity from *nosR-lacZ* fusions containing different lengths of the FixK_2_-like box is indicated on the left side of the figure. Cells were cultured for 24 h under low oxygen conditions (0.5% O_2_) with 10 mM KNO_3_. Data expressed as Miller units (MU) represent mean values and error bars from triplicate samples from at least two independent cultures.

In order to map transcription initiation within the *nosR* promoter region, we identified their Transcriptional Start Sites (TSS) by using 5′-RACE. As shown in **Figure 2A**, we identify two TSS (TSS_1_ and TSS_2_) that initiate at a G and T, 84 and 57 bp upstream of the putative translational start codon, respectively. Analysis of the 5′ region of *nosR* revealed the presence of a purine-rich Shine-Dalgarno-like sequence (GAGG) four bases in front of the *nosR* putative translational start codon. Exhaustive inspection of the *nosR* promoter region failed to identify any putative conserved -35/-10- or -24/-12-type elements associated to σ^70^-dependent or σ^54^-dependent promoters. However, we noticed the presence of an imperfect palindromic sequence (TTGATCCAGCGCAA) positioned at 40.5 and 67.5 bp from TSS_1_ and TSS_2_, respectively (**Figure 2A**). This sequence resembles reasonably well the consensus sequence of the binding site for FixK-type proteins, 5′-TTGA-N_6_-TCAA-3′ (Fischer, 1994; Dufour et al., 2010) and specifically the consensus FixK_2_ binding site [TTG(A/C)-N_6_-(T/G)CAA] recently reported by Bonnet et al. (2013) based on the solved FixK_2_-DNA complex structure.

In order to examine the importance of the FixK_2_-like box identified within the *nosR* promoter region in its transcription, we studied the transcriptional expression derived from a battery of *nosR-lacZ* fusions harboring the full or half FixK_2_-like box, or a deletion of this box (plasmids pBG0304, pBG0305, and pBG0306, respectively) (**Figure 2B** and **Table 1**). These plasmids were integrated into *B. diazoefficiens* WT and β-galactosidase activity was measured in cells cultured under initial 0.5% O_2_ with NO_3_^-^. In contrast to the significant induction of the *nosR-lacZ* transcriptional fusion containing the full FixK_2_-like site, expression of *nosR*-*lacZ* constructs carrying half or deleted FixK_2_-like site was basal, which showed the importance of the presence of this FixK_2_-like binding site in the induction of *nosR* (**Figure 2B**).

### Low Oxygen Is the Main Signal Which Induces Expression of the *nosRZDFYLX* Operon

**FIGURE 3:**
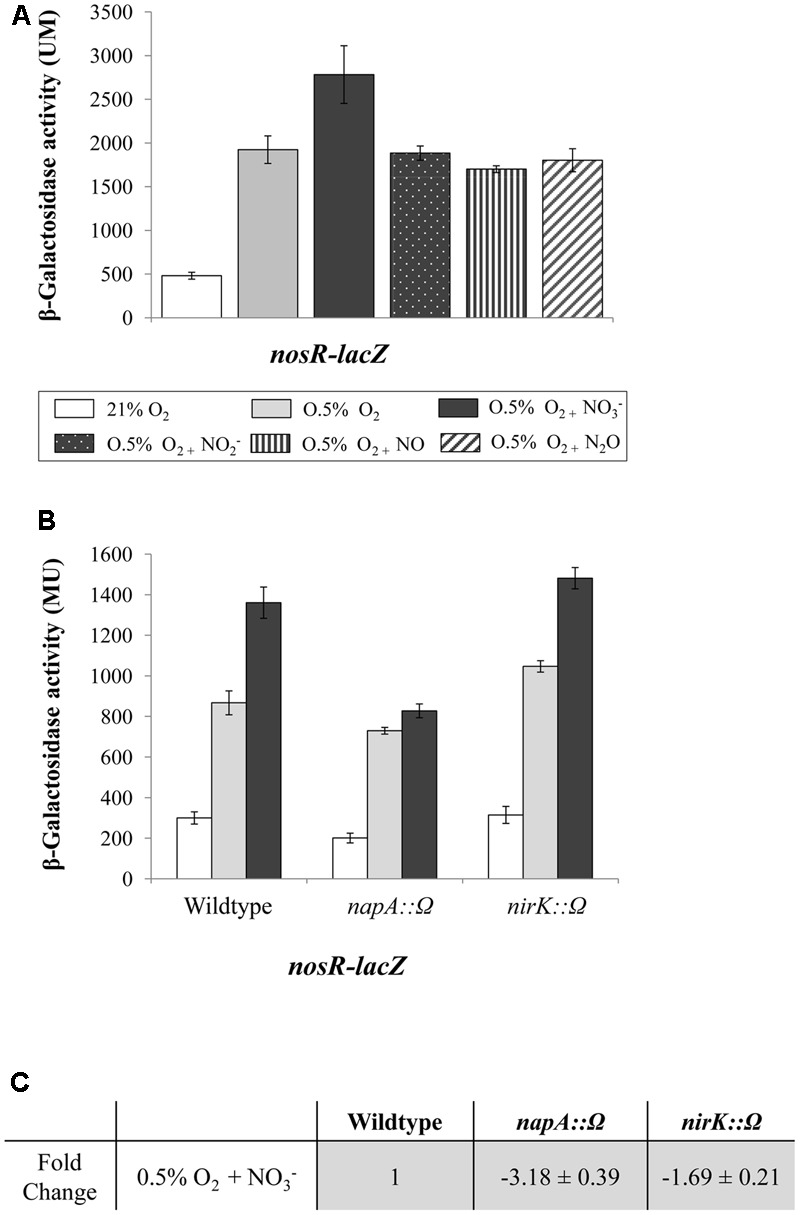
Low-oxygen is the main inducer of *nosRZDFYLX* expression. **(A)** β-Galactosidase activity derived from a *nosR-lacZ* fusion in *B. diazoefficiens* cells grown oxically or under 0.5% O_2_ for 24 h. Then, cells were incubated for another 5 h with or without 10 mM KNO_3_, 500 μM NaNO_2_, 50 μM NO, and 30 mM N_2_O. **(B)** β-Galactosidase activity from the *nosR*-*lacZ* fusion in the *B. diazoefficiens* WT, and mutant strains *napA* and *nirK*. Cells were grown oxically (white bars) or under 0.5% O_2_ in the absence (gray bars) or in the presence of 10 mM KNO_3_ (black bars) during 24 h. **(C)** Expression of *nosR* measured by qRT-PCR. After RNA isolation from cells grown under 0.5% O_2_ in the presence of 10 mM KNO_3_, qRT-PCR reactions were performed with cDNA synthesized from three independent RNA samples assayed in three parallel reactions. Fold-change values refer to differences of expression in the *napA* and *nirK* mutants relative to the WT. In **(A,B)** data expressed as Miller units (MU) are means with standard error bars from at least two independent cultures, assayed in triplicate.

To address the effect of low oxygen and NOx in the expression of the *nosRZDFYLX* operon, we analyzed β-galactosidase activity of the *nosR*-*lacZ* transcriptional fusion in WT cells cultured oxically or under initial 0.5% O_2_, both for 24 h, and later exposed to different NOx (NO_3_^-^, NO_2_^-^, NO, or N_2_O) for additional 5 h-period. As shown in **Figure 3A**, β-galactosidase activity values were basal in cells incubated under oxic conditions. Similar basal levels were observed under oxic conditions in the presence of NO_3_^-^ (data not shown). However, when cells were cultured under 0.5% O_2_, expression of the *nosR*-*lacZ* fusion significantly increased (about fourfold) as compared to oxic conditions (**Figure 3A**). The presence of NO_3_^-^, but not of NO_2_^-^, NO, or N_2_O, slightly increased *nosR*-*lacZ* expression (about 1.5-fold) compared to that observed in cells incubated microoxically in the absence of any NOx (**Figure 3A**).

Next, we were interested to confirm that the lack of NO_3_^-^ reduction products does not affect *nosR*-*lacZ* expression. Therefore, β-galactosidase activity from the *nosR*-*lacZ* fusion was individually analyzed in *napA* or *nirK* mutant strains which are unable to reduce NO_3_^-^ or NO_2_^-^, respectively (Velasco et al., 2001; Delgado et al., 2003). Again, a slight induction of the *nosR*-*lacZ* fusion in the WT cells cultured under 0.5% O_2_ in the presence of NO_3_^-^ was observed (**Figure 3B**), however, no change was detected in the *napA* mutant cultured under the same conditions, suggesting a requirement of NO_3_^-^ reduction on *nosR*-*lacZ* expression. By contrary, induction by NO_3_^-^ of the *nosR*-*lacZ* fusion was retained in the *nirK* mutant indicating that NO_2_^-^ reduction products (NO or N_2_O) are not required for activating the expression of *nosRZDFYLX* genes. These results were validated by qRT-PCR analyses (**Figure 3C**). Similarly as we observed by using the *nosR*-*lacZ* fusion, *nosR* expression was reduced in the *napA* mutant (3.18-fold) compared to WT cells, while it was not significantly affected in the *nirK* mutant (1.69-fold), all cultured in the presence of NO_3_^-^. However, we cannot conclude that the lack of NO_3_^-^-mediated induction of *nos* genes observed in the *napA* mutant (**Figures 3B,C**) is due to the absence of NO_2_^-^, since the addition of NO_2_^-^ to the medium did not increase *nosR-lacZ* expression (**Figure 3A**). Taken together, results from **Figures 3A–C** suggest that microoxia is the main signal that induces expression of *B. diazoefficiens nosRZDFYLX* genes.

### Selective Regulation of *nosRZDFYLX* Genes by FixK_2_ But Not by NnrR

In *B. diazoefficiens*, sensing and transduction of the decrease in O_2_ concentration are mediated by two interlinked O_2_-responsive regulatory cascades, the FixLJ-FixK_2_-NnrR and the RegSR-NifA (Sciotti et al., 2003). A mild decrease in the O_2_ concentration in the gas phase (≤5%) is sufficient to activate expression of FixLJ-FixK_2_-dependent targets, however, a 10-fold lower O_2_ concentration (≤0.5%) is necessary for NifA-mediated activation. In order to investigate how FixK_2_ and NnrR control the microoxic expression of *nosRZDFYLX* genes, we analyzed β-Galactosidase activity from the *nosR-lacZ* fusion in the WT and Δ*fixK_2_* and Δ*nnrR* strains, incubated for 24 h oxically, and microoxically (2% O_2_) in the absence or the presence of 10 mM of KNO_3_. In these experiments, 2% O_2_ concentration was chosen as a middle concentration between 5% (needed for FixLJ-FixK_2_ cascade activation) and 0.5% (required for the activation of the low O_2_-responsive NifA protein), in order to circumvent any possible influence by NifA regulation in our assays.

**FIGURE 4:**
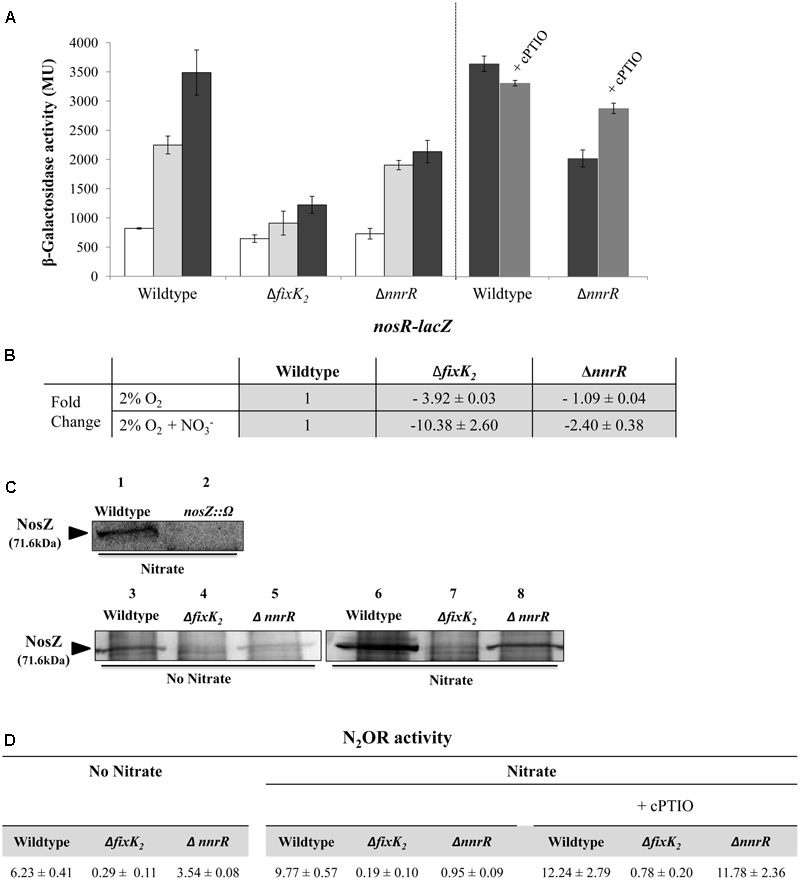
Control of *nosRZDFYLX* expression by the regulatory proteins FixK_2_ and NnrR. **(A)** β-Galactosidase activity expressed as Miller units (MU) from the *nosR-lacZ* transcriptional fusion chromosomally integrated in the *B. diazoefficiens* WT strain, and Δ*nnrR*, and Δ*fixK_2_* strains grown oxically (white bars), under 2% O_2_ in the absence (light gray bars) or in the presence of 10 mM KNO_3_ (black bars) for 24 h. In the right panel, 10 μM of the NO-scavenger cPTIO was added to a series of cultures containing NO_3_^-^ (dark gray bars). **(B)** Expression of *nosR* by qRT-PCR in the WT, and Δ*nnrR*, and Δ*fixK_2_* strains. qRT-PCR reactions were performed with cDNA synthesized from three independent RNA samples assayed in triplicate. Fold-change values refer to differences of expression in the Δ*nnrR*, and Δ*fixK_2_* mutants relative to the WT. **(C)** Western-blotted SDS-PAGE gels of the soluble fraction from the WT and Δ*nnrR*, and Δ*fixK_2_* strains probed with anti-NosZ antibody from *Pa. denitrificans*. As control, a *B. diazoefficens nosZ* mutant was used. The size of *B. diazoefficiens* NosZ is labeled on the left side. **(D)** Nitrous oxide reductase (N_2_OR) activity in the WT and Δ*nnrR*, and Δ*fixK_2_* strains expressed as nmol N_2_O consumed × (mg prot^-1^) h^-1^. In **(B–D)**, cells were grown under 2% O_2_ in the absence or in the presence of 10 mM KNO_3_ during 24 h. 100 μM of cPTIO was added to some of the cultures containing NO_3_^-^ in **(D)**. In **(A,B,D)**, data shown as means with standard errors from at least two independent cultures, assayed in triplicate.

As observed in **Figure 4A**, microoxic induction of *nosR*-*lacZ* was completely abolished in the absence of a functional *fixK_2_* gene, however, it was retained in the Δ*nnrR* strain, suggesting that microoxic expression of *nosRZDFYLX* genes depends on FixK_2_ but not on NnrR. When cells were cultured microoxically in the presence of NO_3_^-^, expression of the *nosR-lacZ* fusion was significantly reduced in the *fixK_2_* mutant (about threefold) compared to that observed in the WT cells (**Figure 4A**). However, β-galactosidase activity of the *nosR*-*lacZ* fusion was slightly reduced in the *nnrR* mutant (about 1.75-fold) compared to the WT (**Figure 4A**). This slight reduction of the expression of the *nosR* gene in the *nnrR* mutant is probably due to the toxic effect of NO that is accumulated in *nnrR* cells as previously reported by Bueno et al. (2017). To check this hypothesis, a NO scavenger (cPTIO) was added during growth of WT and Δ*nnrR* cells under microoxic conditions with NO_3_^-^. As shown in **Figure 4A**, while no effect of cPTIO was observed in WT cells, *nosR-lacZ* expression in Δ*nnrR* cells increased about 40% to that observed in the absence of cPTIO (right panel), which almost corresponds to the expression pattern of the WT. Thus, this indicates that *nos* expression could be partially recovered in the Δ*nnrR* mutant when NO was sequestered by cPTIO.

The different control of *nosR* expression by FixK_2_ or NnrR was also confirmed by qRT-PCR analyses. When cells were cultured microoxically in the absence of NO_3_^-^, expression of *nosR* was reduced in the *fixK_2_* mutant (3.92-fold) compared to that observed in the WT cells (**Figure 4B**), however, it was almost not affected in the *nnrR* mutant (**Figure 4B**). When NO_3_^-^ was added to medium, a significant reduction of *nosR* expression (10.38-fold) was observed in the *fixK*_2_ mutant but only a slight decrease (2.4-fold) was detected in the *nnrR* mutant, both compared to the WT cultured in the same conditions (**Figure 4B**). Taken together, these results suggest FixK_2_ as the transcriptional activator of *nos* genes in response to microoxic conditions.

The differential dependency of *nosRZDFYLX* expression on FixK_2_ and NnrR was also confirmed at protein level by immunoblot analyses using antibodies raised against purified *Pa. denitrificans* NosZ (Felgate et al., 2012). Firstly, we were able to identify NosZ protein in the soluble fraction of *B. diazoefficiens* cells cultured under microoxic conditions (2% O_2_) with NO_3_^-^, since a prominent band of about 70 kDa found in the WT was readily undetectable in the *nosZ* mutant (**Figure 4C**, lanes 1 and 2). The size of this band corresponds to the predicted molecular mass of *B. diazoefficiens* NosZ subunit (71.6 kDa; ProtParam tool^2^). NosZ was already detected in the WT cells cultured microoxically (**Figure 4C**, lane 3) but the presence of NO_3_^-^ slightly increased NosZ steady-state levels (**Figure 4C**, lane 6). This is in line with the observed NO_3_^-^-mediated induction of the *nosR*-*lacZ* fusion (**Figures 3A,B, 4A**). Similarly as the expression pattern observed for the *nosR-lacZ* fusion, NosZ was present in the soluble fraction of Δ*nnrR* cells cultured microoxically either in the absence or in the presence of nitrate (**Figure 4C**, lanes 5 and 8), although at a slightly lower concentration than in the WT cells. As expected, the band of about 70 kDa corresponding to NosZ was absent in the soluble fractions of the Δ*fixK*_2_, independently of the presence or absence of NO_3_^-^ in the incubation medium (**Figure 4C**, lanes 4 and 7).

Finally, we determined N_2_O reductase (N_2_OR) activity in *B. diazoefficiens* WT and *fixK_2_* and *nnrR* mutant strains as the capacity to reduce a defined initial N_2_O concentration. As shown in **Figure 4D**, values of N_2_OR activity in WT cells correlated with NosZ steady-state levels in *B. diazoefficiens* cells (**Figure 4C**), where a slight induction (about 1.6-fold) of activity was observed in the WT cells in the presence of NO_3_^-^ (**Figure 4D**) compared to that observed in exclusively microoxic conditions. In line with the expression pattern of the *nosR*-*lacZ* fusion (**Figure 4A**), *nosR* expression (**Figure 4B**) and NosZ detection (**Figure 4C**, lanes 4 and 7), N_2_OR activity was severely impaired in the Δ*fixK_2_* strain cultivated microoxically independently of the presence of NO_3_^-^ (**Figure 4D**). Under microoxic conditions, cells of the Δ*nnrR* strain showed a milder decrease of N_2_OR activity (about 1.75-fold) compared to that observed in WT cells (**Figure 4D**), which was significantly diminished further (about 10-fold) in the presence of NO_3_^-^ (**Figure 4D**). As we have mentioned above, this strong decrease is probably due to the higher NO accumulation capacity of Δ*nnrR* cells grown microoxically with nitrate compared to WT cells grown under the same conditions (Bueno et al., 2017). In fact, when cPTIO was added during growth, Δ*nnrR* cells restored its ability to reduce N_2_O reaching WT N_2_OR activity values (**Figure 4D**). These data discard the involvement of NnrR as direct regulator of *nos* expression and suggest that the incapacity of Δ*nnrR* to reduce N_2_O under microoxic conditions with NO_3_^-^ is probably due to the accumulation of NO. Taken together, these results pointed out that FixK_2_ is the key transcriptional regulator involved in *nosRZDFYLX* expression.

### The *nosRZDFYLX* Operon Is a Novel Direct Target of FixK_2_

**FIGURE 5:**
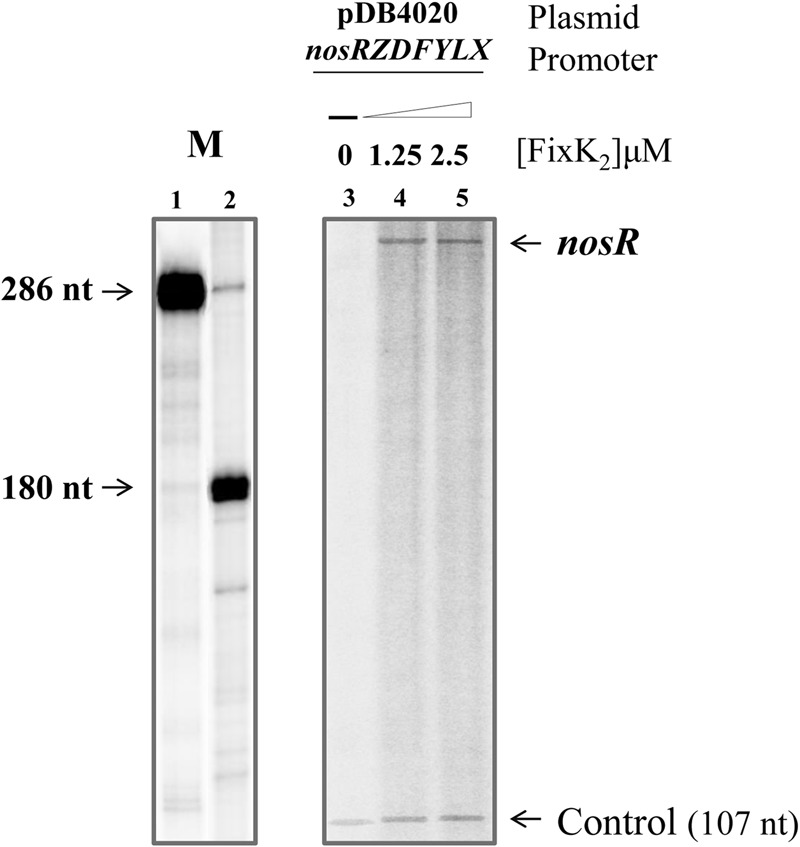
FixK_2_-mediated IVT activation from the *nosRZDFYLX* promoter. pDB4020 plasmid containing the *nosR* promoter cloned upstream of the *rrrn* terminator was used for multiple-round IVT assays with different amounts of purified FixK_2_ protein and RNAP from *B. diazoefficiens*. FixK_2_ concentrations were as follows: no protein, 1.25 and 2.5 μM (lanes 3–5). Transcripts synthesized *in vitro* in the presence of [α-^32^P]UTP were separated on a 6% denaturing polyacrylamide gel and visualized by phosphorimager analysis of the dried gel. Markers transcripts (M) of 286 and 180 nucleotides loaded in lanes 1 and 2 were produced as described by Mesa et al. (2005). The 107-nucleotide transcript present in all lanes originates from a promoter located on the plasmid vector and serves as internal reference. Shown are the results from a transcription experiment that was repeated at least once. Both panels correspond to the same gel. nt, nucleotides.

In order to investigate whether FixK_2_ could have a direct role on *nosRZDFYLX* activation, we monitored RNA synthesis by multiple-round IVT. The *nosR* promoter region was cloned into the template plasmid pRJ9519 (Beck et al., 1997), which carries an *rrn* terminator, yielding plasmid pDB4020. In these experiments, purified C183S-FixK_2_-His_6_ (Bonnet et al., 2013), hereafter referred as FixK_2_, and RNA polymerase (RNAP) holoenzyme from *B. diazoefficiens* that was purified in this work (see Material and Methods) were used. In the absence of FixK_2_, *B. diazoefficiens* RNAP was unable to transcribe the *nosR* promoter efficiently (**Figure 5**, lane 3), whereas it produced a vector-encoded transcript that served as an internal reference. In the presence of FixK_2_ (1.25 and 2.5 μM dimer), *B. diazoefficiens* RNAP transcribed the *nosRZDFYLX* promoter producing a single specific transcript larger than 286 nucleotides (**Figure 5**, lanes 4 and 5, respectively), which probably initiate at TSS_1_. This suggested that the *nosR* promoter is directly activated by FixK_2_ and that transcription from TSS_1_ depends on FixK_2_, at least, in *in vitro* conditions.

## Discussion

Given the damaging effect on climate change of the powerful GHG N_2_O, strategies to mitigate their emissions have to be developed in order to increase agricultural efficiency and decrease current levels of N_2_O production, to satisfy the demands of continuing population growth (Richardson et al., 2009; Thomson et al., 2012). These strategies should include a better understanding of the environmental and molecular factors that contribute to the biological generation and consumption of N_2_O. *B. diazoefficiens*, the endosymbiont of soybeans, contributes to N_2_O emissions given its capacity to carry out the denitrification process under both free-living and symbiotic conditions. Despite the significant knowledge available in this rhizobial species on the regulation of the three first enzymes of denitrification (Nap, NirK, and cNor) involved in N_2_O production (Bueno et al., 2017), the regulatory mechanisms involved in the control of the key step in N_2_O mitigation (the reduction of N_2_O to N_2_) in response to low oxygen and NOx has not been covered in detail. Previous studies have demonstrated that expression of a *nosZ*-*lacZ* fusion depends on low O_2_, the presence of NO_3_^-^ and the FixLJ, FixK_2_ and NosR regulatory proteins (Velasco et al., 2004). The capacity of *B. diazoefficiens* to couple N_2_O reduction to growth as well as a role for the NasST regulatory system on modulation of *nosZ* gene transcription has also been reported (Sánchez et al., 2013, 2014). Furthermore, recent studies have demonstrated the capacity of NasT to interact with *B. diazoefficiens nosR* 5′-leader RNA (Sánchez et al., 2017).

In this work, we have dissected, for the first time, the transcriptional organization of the *nosRZDFYLX* genes in *B. diazoefficiens.* By using RT-PCR we found that the *nosRZDFYLX* genes are transcribed as a single polycistronic mRNA and thus, they are organized as an operon. The transcriptional arrangement of the *nos* genes in other denitrifiers indicate the existence of a diversity of transcriptionally active promoters detected across the *nos* genes between different bacterial species (Zumft and Kroneck, 2007). Supporting our findings, the *Ps. aeruginosa nos* genes are arranged in a single hexacistronic *nosRZDFYL* operon (Arai et al., 2013). A single *nosZ* transcript was identified in *Ps. fluorescens* as well (Philippot et al., 2001). However, in *Ps. stutzeri* three units of monocistronic *nosR* and *nosZ*, and the *nosDFYLtatE* operon (Cuypers et al., 1992; Vollack and Zumft, 2001; Honisch and Zumft, 2003) have been proposed. Similarly, the transcriptional organization of the *nos* cluster of both *Ensifer meliloti* and *Pa. denitrificans* comprises three transcripts: *nosR, nosZ*, and *nosDF*(*Y*), and *nosCR, nosZ*, and *nosDFYLX*, respectively (Holloway et al., 1996; van Spanning, 2011). In order to confirm the results obtained by RT-PCR, we looked for transcriptionally active promoters within *B. diazoefficiens nosRZDFYLX* operon analyzing the transcriptional strength driven by the DNA regions upstream to the *nosR, nosZ*, and *nosD* genes. Interestingly, the highest transcriptional activity was derived from the DNA region upstream of the *nosR* gene compared to that detected from the *nosZ* gene, and no transcription was observed from the 5′ DNA region of the *nosD* gene. The presence of a transcriptionally active promoter upstream of the *nosZ* gene was previously demonstrated by using a *nosZ-lacZ* transcriptional fusion (Velasco et al., 2004) and by performing 5′-RACE (Sánchez et al., 2017). However, since a binding motif for FixK-type regulators was only present within the promoter region of *nosR*, we suggest that this promoter plays the major role in *B. diazoefficiens nosRZDFYLX* regulation.

In this work, we have identified two *nosR* TSS, i.e., TSS_1_ and TSS_2_, positioned at +40.5 and +67.5 bp, respectively, from the axis of symmetry of the FixK-like binding site (TTGATCCAGCGCAA). Similarly, a TSS at +40.5 from the axis of symmetry of the FixK box has been recently identified by Sánchez et al. (2017). In contrast to our results, the TSS at +67.5 bp was not identified in the latter studies. This discrepancy could be due to the different growth conditions used by Sánchez et al. (2017) where cells were cultured in HMM medium (Sameshima-Saito et al., 2006) under anoxic conditions (replacement of O_2_ by N_2_ in the gas phase). FixK_2_-like boxes are present within the promoters of the *B. diazoefficiens napEDABC* (TTGATCCAGATCAA), *nirK* (TTGTTGCAGCGCAA), and *norCBDQD* (TTGCGCCCTGACAA) genes (Velasco et al., 2001; Mesa et al., 2002; Delgado et al., 2003; Supplementary Figure S1). Interestingly, only the *napEDABC*-associated FixK_2_ box as well as the *nosR*-box identified in this work, matches quite well with the consensus FixK_2_ box, TTG(A/C)-N_6_-(T/G)CAA (Mesa et al., 2008, 2009; Bonnet et al., 2013; Supplementary Figure S1). Deletion of this FixK_2_-like box resulted in the complete shutdown of *nosR-lacZ* expression, indicating its essential role in the transcription of the *nosRZDFYLX* operon.

Cells of *B. diazoefficiens* grown oxically showed a basal expression of the *nosR-lacZ* fusion. In this regard, previous observations showed that the *Ps. stutzeri nosZ* gene can also be expressed at high O_2_ concentrations (Miyahara et al., 2010). Supporting these findings, it was recently demonstrated the capacity of both *Ps. stutzeri* and *Pa. denitrificans* to reduce N_2_O under oxic conditions (Desloover et al., 2014; Qu et al., 2015).

Similarly as described for *napEDABC* genes (Bueno et al., 2017), we found that microoxia is sufficient to induce expression of the *nosR-lacZ* fusion, NosZ levels as well as N_2_OR activity. In contrast to that observed for *nosR*/NosZ expression and activity, previous results reported that microoxic expression of *B. diazoefficiens norCBQD* genes required the presence of either NO_3_^-^, NO_2_^-^, or NO, the latter being the signal molecule involved in such control (Bueno et al., 2017). The slight induction of the *nosR-lacZ* fusion in WT cells cultured in the presence of NO_3_^-^ was not observed in cells of a *napA* mutant which does not reduce NO_3_^-^. However, results from **Figure 3A** suggest that any of the NOx derived from NO_3_^-^ reduction (NO_2_^-^, NO, or N_2_O) are not inducers of *nosR-lacZ* expression. Furthermore, NO is not required for *nosR-lacZ* induction, since WT levels of *nosR* expression were observed in a *nirK* mutant which does not reduce NO_2_^-^ to NO. Likewise as we found in this work, previous studies suggested N_2_O as a weak inducer of *nosZ* genes in several bacteria (Kroneck et al., 1989; Richardson et al., 1991; Sabaty et al., 1999). Taken together, these observations suggest a very mild effect of NOx in the expression of *nos* genes. Therefore, it might be possible that a change in the cellular redox state derived from NO_3_^-^ reduction by Nap is involved in *nosR*-*lacZ* induction. In fact, our own previous results demonstrated the involvement of the *B. diazoefficiens* redox-responsive regulatory protein RegR on the expression of *nos* genes (Torres et al., 2014). Alternatively, the NasST system might be involved in the NO_3_^-^-mediated response of *nos* genes expression (Sánchez et al., 2014).

Microoxic induction of the *nosRZDFYLX* genes as well as NosZ expression in *B. diazoefficiens* depends on FixK_2_, but not on NnrR. The dependency of *nosRZDFYLX* transcription on FixK_2_ was demonstrated by IVT transcription experiments carried out with oxically purified protein in collaboration with *B. diazoefficiens* RNAP. In the same manner, microoxic induction of the *B. diazoefficiens napEDABC* genes depends on FixK_2_, but not on NnrR, probably due to its NOx-independent expression (Bueno et al., 2017). In fact, FixK_2_ also activates transcription of *napEDABC* genes (Bueno et al., 2017).

In contrast to our results, NO has been proposed as the signal that upregulates the *nosR, nosZ*, and *nosD* promoters in *Ps. aeruginosa, Ps. stutzeri*, and *Pa. denitrificans* (reviewed by Zumft and Kroneck, 2007). In *Rhodobacter sphaeroides* IL106 *nosZ* expression depends on one of the reduction products of NO_3_^-^, suggesting NO as the signal molecule, too (Sabaty et al., 1999). Further, global gene expression analysis carried out with *E*. *meliloti* showed induction of *nos* genes in response to NO (Meilhoc et al., 2010). NO-dependent induction of *nos* genes in *Ps. aeruginosa, Ps. Stutzeri*, or *Pa. denitrificans* is processed via the regulatory proteins DNR/DnrD/NNR, respectively (van Spanning et al., 1999; Vollack and Zumft, 2001; Arai et al., 2013). While *Ps. aeruginosa* DNR is under the control of the low O_2_-sensing protein ANR (Trunk et al., 2010), transcription of *dnrD* in *Ps. stutzeri* is activated in cells grown under O_2_ limitation conditions, being particularly strong in denitrifying cells, but not under the control of the low-O_2_ sensor FnrA (Vollack et al., 1999). A particular case constitutes *Pa. denitrificans*, where N_2_O reduction is subjected to a robust regulation by FnrP and NNR in response to low oxygen (via FnrP) or NO (via NNR) (Bergaust et al., 2012).

The reduced induction of *nosR/*NosZ expression observed in Δ*nnrR* cells cultured with NO_3_^-^ that has also been described previously for *napEDABC* genes expression (Bueno et al., 2017), might be a consequence of the higher capacity to accumulate NO by the *nnrR* mutant strain compared to the WT strain (Bueno et al., 2017). Supporting this hypothesis, when NO was removed by adding the NO-scavenger cPTIO to the Δ*nnrR* cultures with NO_3_^-^, *nosR-lacZ* expression as well as N_2_OR activity restored to WT levels. It might be possible that the NosZ catalytic center Cu_z_ which remains in a redox-inert, paramagnetic state Cu_z_^∗^ (Wunsch and Zumft, 2005), is inactivated in the presence of NO accumulated by the *nnrR* mutant (Dell’Acqua et al., 2011). However, the precise mechanism involved in NosZ inactivation by NO is still unknown.

This work performed with the model rhizobial denitrifier *B. diazoefficiens* expands the understanding of the environmental and regulatory factors involved in the reduction of N_2_O, the key step that mitigates N_2_O emissions. We hope that our results would help to establish action plans for the development of practical strategies for mitigation of N_2_O emissions from legume crops.

## Author Contributions

MT, EB, MD, and SM conceived and designed the study. MT, EB, AJ-L, and JC performed the experiments. MT, EB, AJ-L, JC, MD, and SM analyzed the results. MT, EB, MD, and SM wrote the manuscript. EB critically revised the manuscript. All authors read and approved the final manuscript.

## Conflict of Interest Statement

The authors declare that the research was conducted in the absence of any commercial or financial relationships that could be construed as a potential conflict of interest.

## Footnotes

1 http://pallab.serc.iisc.ernet.in/gester/dbsearch.php

2 http://web.expasy.org/protparam/
