# Supplementary material for: FixK2 Is the Main Transcriptional Activator of Bradyrhizobium diazoefficiens nosRZDYFLX Genes in Response to Low Oxygen
Source: Front Microbiol. 2017 Aug 30;8:1621. doi: 10.3389/fmicb.2017.01621 (PMC5582078; doi:10.3389/fmicb.2017.01621)
Supplement: Supplementary file 1 [file Data_Sheet_1.DOCX]

**Supporting information**

| **Table S1.** Oligonucleotide primers used in this study. | | | | |
| --- | --- | --- | --- | --- |
| **Primer** | **DNA sequence (5’ → 3’)** | | | |
| **RT-PCR co-transcription** | | |  | |
|  | |  | | |
| a1 | | CGTAGGACCAGAATGCGAAGG | | |
| a2 | | CGACAGGTAACCGTTCAGG | | |
| b1 | | TTCTGCGGTTGGCTCTG | | |
| b2 | | TTTGTTACCACCGCCCC | | |
| c1 | | CGGCCTACGACGGCAAGGG | | |
| c2 | | CAGCAGTCAGCGTCTCC | | |
| d1 | | GGAGGGCGGAAGATGAG | | |
| d2 | | GGAGTTGCGAGATCGAG | | |
| e1 | | CTCGATCTCGCAACTCC | | |
| e2 | | ATTGAGGAACAGCAGCG | | |
| f1 | | CGCTGCTGTTCCTCAAT | | |
| f2 | | CCTGCTTGCGGCTCTCG | | |
| g1 | | CGAGAGCCGCAAGCAGG | | |
| g2 | | CTGCTGCCCACCTTCGC | | |
| h1 | | GGCACCAGCAAATCTCA | | |
| h2 | | CTTGGAAAGCGTGTGTGCG | | |
|  | |  | | |
| **qRT-PCR** | |  | | |
|  | |  | | |
| nosR_qRT_PCR_F | | ATGATCCAGGTGCGGCTGAAG | | |
| nosR_qRT_PCR_R | | CCGGCTGTGATGATTGTGTTCG | | |
| 16S_qRT_For | | GCAGGCTTAACACATGCAAGTC | | |
| 16S_qRT_Rev | | AGGTACGTTCCCACGCGTTACTC | | |
|  | |  | | |
| **5’RACE** | |  | | |
|  | |  | | |
| SP1_nosR | | GGAATAGCCGACCGCATTGG | | |
| SP2_ nosR | | GCGTCTAGAGACGCGGTGTTGAAGGACGAG^(*)^ | | |
| (dT)_17_-adaptor-primer | | GACTCGAGTCGACATCGATTTTTTTTTTTTTTTTT | | |
| adaptor-primer | | GACTCGAGTCGACATCG^(*)^ | | |
| SP6 | | ATTTAGGTGACACTATAG | | |
|  | |  | | |
| **Transcriptional fusions** | | | |  |
|  |  | | | |
| PnosR.r | GGGACCTCGAACGTGAAC | | | |
| PnosZ.f | GGATGCGGATATGGCGCG | | | |
| PnosZ.r | GATTGCCTCCTCGGCTTTCG | | | |
| PnosRfull.f | CTTGATCCAGCGCAAACACTC | | | |
| PnosRhalf.f | ATCCAGCGCAAACACTCTTTCG | | | |
| PnosRno.f | CCTGCGTCAACGGCGACTTC | | | |
|  |  | | | |
| **IVT template** |  | | | |
| nosR_For_Transc | AAATCTAGAGGCGCGATTGTGACCATCTTGC^(*)^ | | | |
| nosR_Rev_Transc | AAAGAATTCGGACGAGAGCACGCAAAGGAAC^(*)^ | | | |

^(*)^ Engineered XbaI, XhoI, SalI and EcoRI restriction sites are underlined.

**Figure S1:**


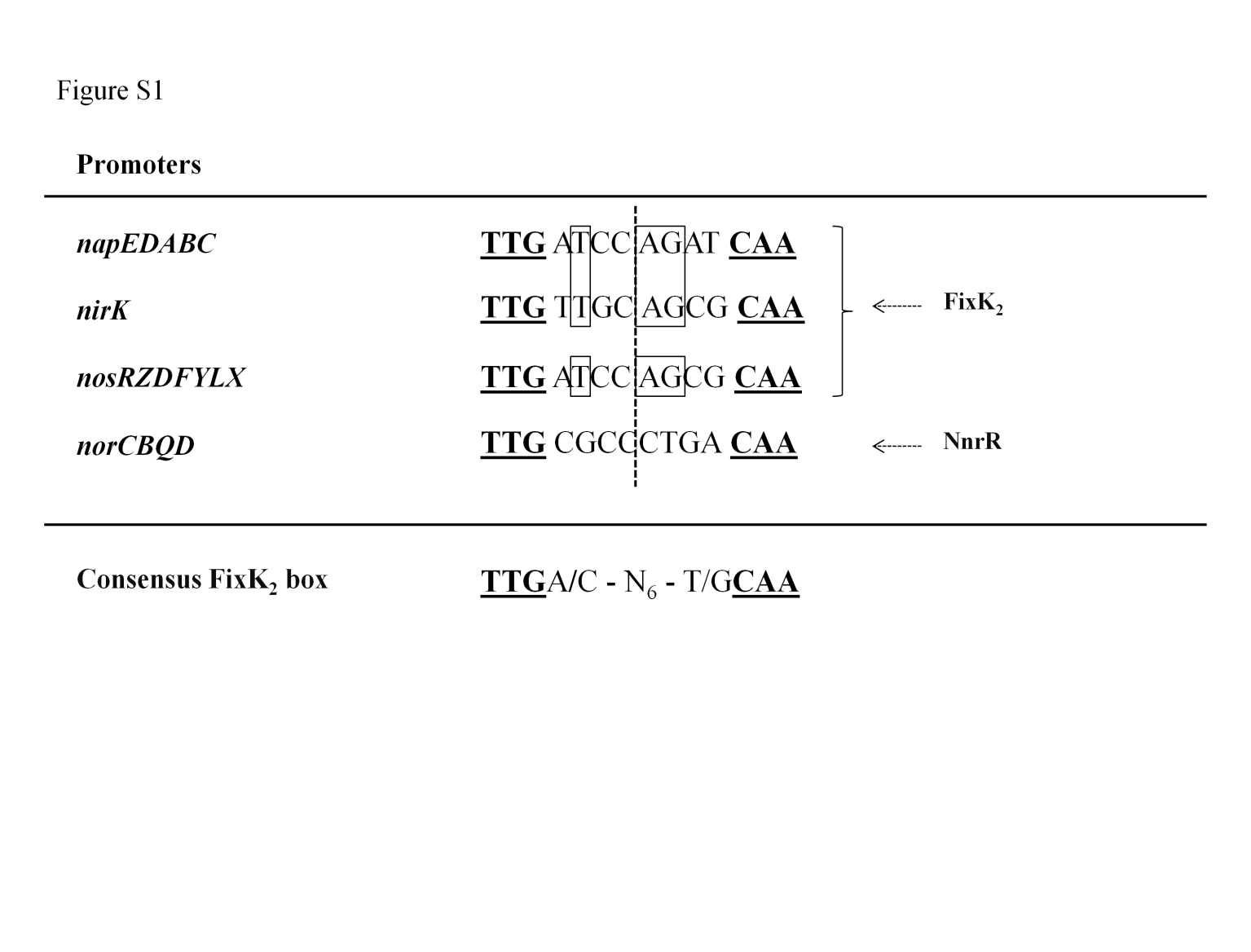


**Figure S1.** FixK_2_-like boxes associated with the promoters of *B. diazoefficiens* *napEDABC*, *nirK*, *norCBQD* and *nosRZDYFLX* denitrifying genes. The FixK_2_ consensus binding site as described by Bonnet and coworkers (2013) is shown at the bottom of the figure. Note that the FixK_2_ binding sites present at *napEDABC* and *nosRZDYFLX* promoters perfectly match with this consensus. Letters inside squares show the conserved determinants common for *napEDABC-*, *nirK*- and *nosRZDYFLX*- compared with *norCBQD*-associated FixK_2_-like boxes. The vertical dotted line marks a dyad symmetry axis with respect to the most conserved nucleotides, indicated by bold style and underlined. The arrows indicate the approved interaction of FixK_2_ and NnrR with the specified FixK_2_-like binding sites.

**References**

Bonnet, M., Kurz, M., Mesa, S., Briand, C., Hennecke, H., and Grutter, M.G. (2013) The structure of *Bradyrhizobium japonicum* transcription factor FixK_2_ unveils sites of DNA binding and oxidation. *J Biol Chem* **288**: 14238-14246.
